# Prophage-Mediated Dynamics of ‘*Candidatus* Liberibacter asiaticus’ Populations, the Destructive Bacterial Pathogens of Citrus Huanglongbing

**DOI:** 10.1371/journal.pone.0082248

**Published:** 2013-12-13

**Authors:** Lijuan Zhou, Charles A. Powell, Wenbin Li, Mike Irey, Yongping Duan

**Affiliations:** 1 Horticultural Research Laboratory, Agricultural Research Service, US Department of Agriculture, Fort Pierce, Florida, United States of America; 2 Indian River Research and Education Center, University of Florida, Fort Pierce, Florida, United States of America; 3 Center for Plant Health, Science and Technology, Animal and Plant health Inspection Service, US Department of Agriculture, Beltsville, Maryland, United States of America; 4 Southern Garden Citrus, U. S. Sugar Corp., Clewiston, Florida, United States of America; East Carolina University, United States of America

## Abstract

Prophages are highly dynamic components in the bacterial genome and play an important role in intraspecies variations. There are at least two prophages in the chromosomes of *Candidatus* Liberibacter asiaticus’ (Las) Floridian isolates. Las is both unculturable and the most prevalent species of Liberibacter pathogens that cause huanglongbing (HLB), a worldwide destructive disease of citrus. In this study, seven new prophage variants resulting from two hyper-variable regions were identified by screening clone libraries of infected citrus, periwinkle and psyllids. Among them, Types A and B share highly conserved sequences and localize within the two prophages, FP1 and FP2, respectively. Although Types B and C were abundant in all three libraries, Type A was much more abundant in the libraries from the Las-infected psyllids than from the Las-infected plants, and Type D was only identified in libraries from the infected host plants but not from the infected psyllids. Sequence analysis of these variants revealed that the variations may result from recombination and rearrangement events. Conventional PCR results using type-specific molecular markers indicated that A, B, C and D are the four most abundant types in Las-infected citrus and periwinkle. However, only three types, A, B and C are abundant in Las-infected psyllids. Typing results for Las-infected citrus field samples indicated that mixed populations of Las bacteria present in Floridian isolates, but only the Type D population was correlated with the blotchy mottle symptom. Extended cloning and sequencing of the Type D region revealed a third prophage/phage in the Las genome, which may derive from the recombination of FP1 and FP2. Dramatic variations in these prophage regions were also found among the global Las isolates. These results are the first to demonstrate the prophage/phage-mediated dynamics of Las populations in plant and insect hosts, and their correlation with insect transmission and disease development.

## Introduction

Bacteriophages are the most abundant organisms in the biosphere with an estimated 10^31^ particles [1]. After invading a living bacterial cell, bacteriophages can multiply using bacterial materials and phage enzymes by two different mechanisms, the lytic cycle as phage and the lysogenic cycle as prophage. Phages/prophages have long been used to distinguish bacterial isolates from different environmental and clinical sources [2]–[4]. Based on their lateral gene transfer, phages/prophages are sometimes considered the most dynamic mobile element affecting bacterial pathogenicity, host specificity, and adaptation to individual ecological niches [5]–[8]. Since phages/prophages have no negative effects on eukaryotes, they have been employed in the therapy of various human, animal, and plant diseases [9]–[11].

Insight into prophage/phage biodiversity and their interactions with host genomes was accomplished by the development and application of next generation sequencing and comparative genomics technology. To date, more than one thousand phage genomes are available in the European Bioinformatics Institute (EBI) phage database (http://www.ebi.ac.uk/genomes/phage.html). In addition, the majority of the bacterial genome sequences deposited in the National Center for Biotechnology Information (NCBI) database contains at least one prophage sequence [12]. They are classified into inducible functional prophages and prophage-like elements, such as defective and satellite prophages, or gene transfer agents [13]. Prophage or prophage-like elements have been identified from various plant pathogenic bacteria, such as *Xylella fastidiosa* [14], *Xanthomonas citri* (Xac), *Xanthomonas campestris* pv. *campestris* [15], *Pseudomonas syringae* pv. *Actinidiae* [16], and *Candidatus* Liberibacter asiaticus [17], [18].

“*Ca.* Liberibacter asiaticus” (Las) is one of the three Liberibacter species associated with citrus Huanglongbing (HLB), a century-old and emerging disease that impedes citrus production worldwide [19], [20]. HLB was first noted in China in early 20^th^ century [21], but it has only recently attracted attention in the United States because of its rapid spread throughout Florida since 2005 [22] and recent identification in Texas and California [23]. Another two *Ca.* Liberibacter species associated with HLB are *Ca.* Liberibacter africanus in Africa [24] and *Ca.* Liberibacter americanus in Brazil [25], [26]. The transmission of these phloem-limited *alphaproteobacteria* relies on their insect vector, citrus psyllids (*Diaphorina citri* and *Trioza erytreae*) or on grafting [27]. The Las bacterium has a significantly reduced genome (1.26 Mb). It lacks type III and type IV secretion systems, and free-living or plant-colonizing extracellular degradative enzymes [28]. The Las psy62 genome does not contain any known transposon or insertion sequence (IS) elements, but encompasses multiple prophage or prophage-like elements. Two prophages, FP1 (39,959 bp) and FP2 (38,551 bp) have been identified in the Las psy62 genome [18]. They account for approximately 1/16 of the entire Las genome and were characterized as functional homologues between these two prophages [17].

Various symptoms of HLB can be observed in Las-infected plants, especially the uneven distribution of the disease and the variation in pathogen titers within a single infected plant due to their unique host-pathogen interactions. The typical symptoms of HLB-affected citrus include yellow shoot, vein yellowing, asymmetric blotchy-mottle on leaves, and leaf curl with vein corking. HLB-affected trees may be smaller with upright leaves, have lopsided, bitter fruit and go through early leaf-drop and die-back, and often die in 3–8 years after becoming symptomatic. However, many HLB-affected trees do not show uniform symptoms and may have branches that are free of all symptoms [20]. We hypothesized that the spatial and temporal variations of different Las populations may contribute to the variations of Las bacterial titers and HLB symptoms observed in the infected host plants. In our previous work, two related hyper-variable genes (*hyv*_I_ and *hyv*_II_) with multiple nearly identical tandem-repeats were identified from the two prophage regions [18]. The *hyv*_I_ and *hyv*_II_ genes from global Las isolates have extensive variations in the intragenic repeat numbers, repeat arrangements, and the sequences flanking the repeat region, indicating the extensive diversity of Las populations in the world. These differences were also noted within DNA samples from a single infected tree displaying various symptoms on the different branches, suggesting that the genetic variations in the Las prophage region may play an important role in these host-microbe interactions. Based on Las prophage sequence variations, another locus in the Las prophage region [29] and a prophage terminase gene were identified [30] as mosaic loci to encompass sequence variations among different Las isolates. In this study, we investigated two other highly diversified regions of Las prophages and revealed the prophage/phage-mediated dynamics of the Las populations in plant and insect hosts and their correlation with insect transmission and disease development.

## Materials and Methods

### Las Bacterial Source

A total of 215 Las-infected citrus field samples were collected in Florida. Among them, 124 citrus samples from the USHRL’s (United States Horticultural Research Laboratory of the US Department of Agriculture) Ft. Pierce farm were collected from 100 citrus plants with different varieties, including sweet orange (*Citrus sinensis*), sour orange (*Citrus aurantium*), tangelo (*Citrus tangelo*), mandarin (*Citrus reticulata*), grapefruit (*Citrus paradisi*), pomelo (*Citrus maxima*), lemon (*Citrus limon*) and trifoliate orange (*Poncirus trifoliata*). Two types of samples were collected from some trees with particular symptoms, one displaying typical blotchy mottle and the other displaying vein yellowing and/or nutrition deficiency-like symptoms. The other group of 91 citrus samples were collected from commercial citrus groves in 16 counties of Florida from 2006–2008 (Figure S1). In addition, 42 HLB-affected periwinkle samples containing dodder-transmitted or graft-transmitted Las were collected from the USHRL’s insect-proof greenhouse where they were maintained from 2007–2012 as described previously [18]. 129 Las-infected psyllids were collected from HLB-affected citrus plants at the USHRL’s Ft. Pierce farm or raised in the USHRL’s insectary using Las-infected plants containing positive Type D population from 2009–2012. DNA samples from Las-infected plant or psyllids from China, Brazil, the Philippines and Thailand were kindly provided by our collaborators.

### DNA Extraction and Las Bacterial Titration

Total DNA was extracted from leaf midribs following the DNeasy® Plant Mini Kit standard protocols (Qiagen Inc., Valencia, CA) [31], or using the PVP method as describe previously [18]. Total DNA was extracted from single Asian citrus psyllid (ACP) by the phenol/chloroform method [28]. All the plant or ACP DNA samples were subjected to Las titer estimation by quantitative real-time PCR using 16S rDNA-based primers and probe [32]. Plant or ACP samples producing real-time Ct values between 15 and 26 were used for the identification of different types and typing analysis.

### Las Prophage Library Construction

Primers used in this study were designed using Oligo 7.23 (Molecular Biology Insights, Inc., Cascade, CO, USA). Primers LJ753/LJ759 (Table 1) were used to amplify the hyper-variable region (HVR) of the Las prophage genome. Conventional PCR was carried out in a 20 µL volume, which contained one unit of High Fidelity Platinum® Taq DNA Polymerase (Invitrogen, Carlsbad, CA, USA), 1× High Fidelity PCR buffer, 0.2 mM each dNTP, 2.0 mM MgSO_4_, 250 nM forward/reverse primer and 1–2 µL template DNA. PCR was performed using a program with an initial denaturation at 95°C for 3 min, 38 cycles of 94°C for 20 s, 54°C for 20 s and 68°C for 3 min and a final 10 min extension at 68°C, in a C1000™ Thermal Cycler (Bio-Rad, Hercules, CA). Poly “A” tails were added to the PCR products by adding 0.5 U of regular Taq DNA polymerase (New England BioLabs Inc., Ipswich, MA, USA) and incubating at 72°C for 10 min in a thermocycler. PCR products with poly “A” tails were ligated into the pCR8/GW-TOPO vector and transformed into TOP10 One Shot chemically competent cells according to the manufacturer’s instructions (Invitrogen, Carlsbad, CA, USA). The TOPO clone libraries were generated from Las-infected citrus, periwinkle and psyllid samples, respectively. Recombinant transformants were screened by conventional PCR using vector based M13f and M13r primers. Individual colonies with inserts were grown overnight in 96 well plates (3 plates each) with 1.6 mL LB liquid medium supplemented with 100 mg/µL spectinomycin. Plasmid DNA was extracted from the overnight cultures using the QIAprep 96 Turbo Miniprep kit following the manufacturer’s instructions (QIAGEN, Hilden, Germany).

**Table 1:** Primers used in this study.

Name	Sequence (5′–3′)	Description
LJ753	ACCCAACCGTGAAAAGAAAC	Use for identification of Types A, B, C, E, A1, A2, C1, C2
LJ759	AAACATCCACCCCCGAACC	Use for identification of Types A, B, C, D, E, A1, A2, C1, C2
LJ513	CTTACGCTCTTGGGCTATG	Extend Type D sequence: 5,654 bp
LJ798	CCATTTTCTAGGTCTCCGTAC	
LJ862	ACTTATATCTGGATCTGCGTTC	Extend Type D sequence: 6,846 bp
LJ834	AAGACATTTTAAGACACATTGAG	
LJ799	TGTTACCATAAATACCGTGC	Specific for Type A when used with LJ797
LJ797	TGTTTGATTTCCTTGTAAAAGTC	
LJ898	TTATAAACTTTTACATACGTGAG	Specific for Type B when used with LJ864
LJ864	AACGGGTTAAGGTGGTAGGG	
LJ1262	CGTGGTAACCATTTACGTATT	For Type C when used with LJ850
LJ850	TACTGGGGTTTAGGGCACG	
LJ860	GCTTGCAGAAGAGAAATGTAAG	Specific for Type D when used with LJ863
LJ863	CAACCGCACAAAATATCAAGC	

### Rarefaction Analysis

Rarefaction is a statistical method commonly used in microbial diversity studies of different environmental samples [33]–[36], or human samples [37]. Rarefaction curves were generated in Excell (Microsoft, Redmond, WA) using datasets calculated by the Analytic Rarefaction version 1.3 software developed by Steven Holland (Department of Geology, the University of Georgia [http://strata.uga.edu/software/]). The percentage of coverage was calculated by Good’s method [38] with the formula [1 − (*n*/*N*)] × 100, where *n* is the number of types represented by one clone and *N* is the total number of clones analyzed [37]. The diversity of the sequences in each library was estimated by diversity indices, including species richness and Simpson, using Diversity Index tools in SPADE (Institute of Statistics, National Tsing Hua University [http://chao.stat.nthu.edu.tw/]). Species richness was estimated by a bias-corrected Chao1 (S*chao1-bc*) method and abundance-based coverage estimator (S*_ACE_*) [39], [40].

### Cloning and Sequencing Type D Flanking Region

Sequence alignment of Type D to two of the Las prophages, FP1 and FP2, indicated a rearrangement in the prophage regions. In accordance with the rearranged regions in FP2, two sets of primers LJ513/798 and LJ862/834 (Table 1) were designed and used for sequence extension of the Type D and its flanking region from a Las-infected periwinkle sample. PCR was performed under the same conditions using High Fidelity Platinum® Taq DNA Polymerase as noted above except that the extension time was changed to 6 min at 68°C in each cycle. Cloning, sequencing, assembly and alignment analysis of the long PCR products were carried out as described above. To confirm the extended sequence of the Type D region, long PCR using primers LJ513/834 was carried out under the same conditions described above except the extension time in each cycle was increased to 11 min.

### Typing Las Bacterial Variants

Specific conventional PCR primers for each abundant sequence type were designed (Table 1) and used for analysis of Las variant types in different hosts. The PCR reactions were carried out in a 20 µL volume containing 1 × buffer D (Epicentre Biotechnologies, Madison, WI, USA), 250 nM forward/reverse primer, 1.25 U *Taq* DNA polymerase (New England BioLabs Inc., Ipswich, MA) and 1–2 µL template DNA, using an amplification program of 95°C for 3 min, followed by 38 cycles of 94°C for 20 s, 54–64°C for 20 s depending on the primer set, 72°C for 1–2 min based on the size of the PCR products, and a final elongation step of 7 min at 72°C.

### Sequencing and Sequence Analysis

Sequencing was performed by the Core Genomics Facility at the USHRL using BigDye Terminator version 3.1 and the 3730×l DNA analyzer (Applied Biosystems, Carlsbad, CA). Sequences were assembled by Sequencher 4.10.1 (Gene Codes Corporation, Ann Arbor, MI) and the alignment was conducted using Align X in Vector NTI (Invitrogen, Carlsbad, CA). The open reading frames (ORFs) for each type of sequence were predicted using GeneMark [41]. The remote homology detection server HHpred [42] was used to search for homologues of predicted proteins from each type of sequence in the Protein Data Bank with default parameters. DNA secondary structures were predicted by Fold DNA Single Strain in RNAstructure software (Version 5.4, Mathews Lab, University of Rochester Medical Center [http://rna.urmc.rochester.edu/RNAstructure.html]).

### Nucleotide Sequence Accession Numbers

The annotated 9 different types of nucleotide sequences in Las prophage HVR have been deposited in the GenBank under accession numbers JX275489–JX275497 as in Table 2.

**Table 2:** Different types of sequences identified from Las-infected plant and psyllid hosts based on the hyper-variable region of the prophages in the genome.

Type name	Identification primers	Length (bp)	Percentage in library (%)			Specific primers	Assigned Accession number
			psyllid	periwinkle	citrus		
A	LJ753/LJ759	3107	36.71	0.36	20.14	LJ799/LJ858	JX275489
B	LJ753/LJ759	2988	25.17	43.73	64.24	LJ898/LJ864	JX275490
C	LJ753/LJ759	2692	29.72	39.07	11.11	LJ1262/LJ850	JX275491
D	LJ759	2123	0	9.32	1.39	LJ860/LJ863	JX275492*
E	LJ753/LJ759	3072	0	0.72	0.35	LJ1237/LJ854	JX275493
A1	LJ753/LJ759	2747	1.05	0.36	0.35	LJ1269/LJ850	JX275494
A2	LJ753/LJ759	2728	0.35	0.36	0	LJ1262/LJ1266	JX275495
C1	LJ753/LJ759	2682	4.90	2.15	0.35	/	JX275496
C2	LJ753/LJ759	2703	2.10	3.94	2.08	/	JX275497

The Type D sequence deposited in GenBank was an 11,121 bp fragment extended from the 2,123 bp sequence amplified by the invert repeat primer LJ759.

## Results

### Las Prophage Types and Distribution

**Figure 1:**
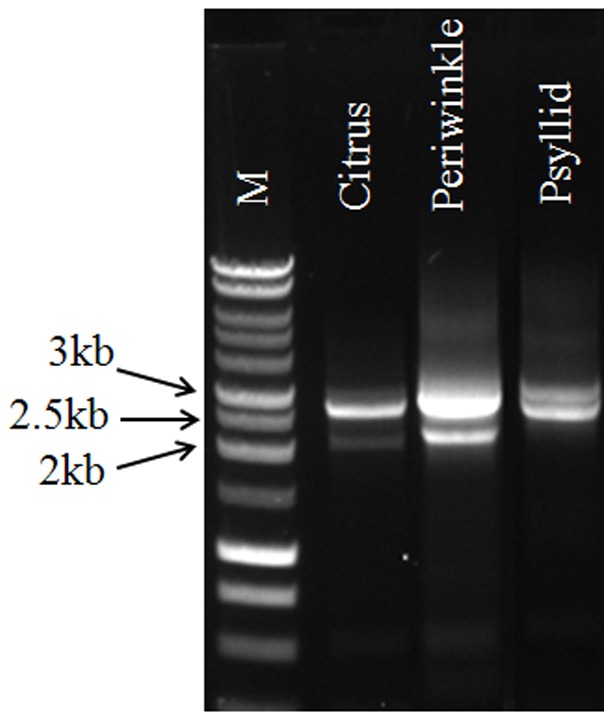
PCR products amplified from Las positive citrus, periwinkle and psyllid samples using primer LJ793/LJ795. M: 1 kb DNA ladder (Promega, Madison, USA); The CT value for three isolates by Taqman real-time PCR using 16 s rRNA gene based primers are: citrus, 19.95; periwinkle, 18.58; and psyllid, 18.26.

**Figure 2:**
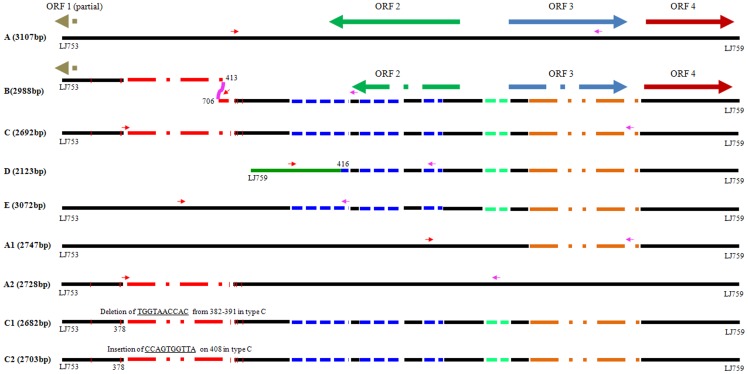
Schematic diagram of nine types of sequences identified from Las–infected citrus, periwinkle and psyllid. Lines of the same color are conserved regions among each type; long dashes and dots in the same color represent the deleted sequences and the rectangles in the same color represent the inserted sequences in this region. Arrows of different colors are predicted ORFs, same color arrows with long dashes and dots are homologous proteins with deletions; small arrows in red or pink are forward and reverse primers specific for each sequence type.

Multi-band PCR products were obtained from Las-infected citrus, periwinkle and psyllid samples using primers LJ753/LJ759 (Figure 1), and a total of nine sequence types were identified from these three cloning libraries (Table 2). Four dominant types of sequences accounted for more than 90% of each library, and they were designated as Types A, B, C, and D corresponding to sizes 3,107 bp, 2,988 bp, 2,692 bp and 2,123 bp, respectively. The Type A sequence was located in prophage FP1 (39,959 bp, CP001677.5) and the Type B sequence in prophage FP2 (38,551 bp, JF773396.1). Type C was a variant of Type B with a deletion from 412 to 706 bp (Figure 2). The Type D sequences were amplified from citrus and periwinkle isolates by invert repeat primer LJ759, and the first 415 bp shared 99.9% similarity with an inverted downstream sequence outside of the LJ753/LJ759 region in psy62-FP2 (Figure 2). Types E, A1 and A2 were chimeric like sequences derived from the homologous recombination between Types A and B or Types A and C (Figure 2). Types C1 and C2 sequences were highly similar to the Type C sequence. Ten bp deletions at position 382–391 and 408–417 in Type C resulted in Type C1 and Type C2, respectively.

The most abundant type in the psyllid library was Type A (36.71%), followed by Type B (25.17%) and then Type C (19.72%). Types A1, A2, C1 and C2 were rare (8.4% in total) in the psyllid library, and neither Type D nor E was present in the 286 psyllid clones (Table 2). Interestingly, the Type A sequence was very rare (0.36%) in the screened 278 periwinkle clones even though a citrus source containing Type A was used to inoculate the periwinkle plants. The most abundant type in the periwinkle library was Type B (43.73%) followed by Type C (39.07%) and then Type D (9.32%). Type B was also the most abundant type (64.24%) in the citrus library, followed by Type A (20.14%), C (11.11%) and D (1.39%). Types E, A1, A2, C1 and C2 are rare in the citrus (3.13% in total) and periwinkle libraries (7.53% in total), and Type A2 was not identified from the screened 288 citrus clones (Table 2).

### Las Prophage Diversity

**Figure 3:**
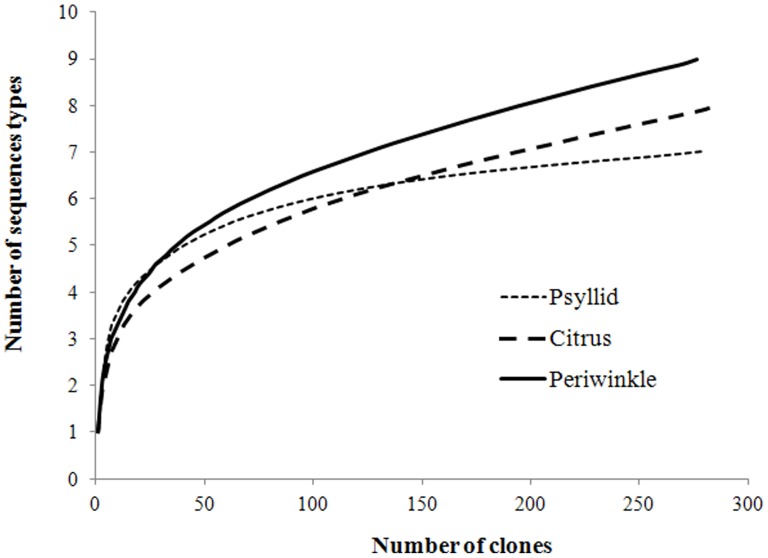
Rarefaction analysis for the number of different types of sequences in three clone libraries.

Rarefaction analysis was applied to compare the species richness and evaluate if the sample size was sufficient to estimate the diversity level in three TOPO clone libraries. The values of Good’s coverage were around 99% for the citrus, periwinkle and psyllid libraries. This showed that the number of the randomly picked clones was sufficient to identify diversity in the clone libraries (Table 3). Based on the rarefaction curve established by plotting the number of different types of sequences versus the number of clones used from three libraries (Figure 3), periwinkle harbored a higher level of diversity in the prophage HVR than citrus or psyllid. The calculated rarefaction curve approached saturation for the psyllid library, indicating that the diversity of HVR sequences in the psyllid clone library was almost fully covered. However, the steep slopes and the lack of plateau for rarefaction curves created from citrus and periwinkle libraries indicated that further sampling of these clone libraries likely would have revealed additional variant(s). These results were supported by the differences in species richness and Simpson indices (Table 3).

**Table 3:** Diversity indices and library coverage estimations.

	No. ofclones	Coverage	S*_Chao1-bc_*	S*_ACE_*	Simpson
Citrus	288	99.0%	11.0	12.4	0.464
Periwinkle	278	98.9%	10.5	15.0	0.352
Psyllid	286	99.7%	7.0	7.7	0.287

*_Chao1-bc_* and S*_ACE_* : Species richness using the Chao-bc method and Abundance-based Coverage Estimator (ACE) method, respectively. Simpson index was estimated by the maximum likelihood estimator (MLE). S

### Type Variations of Las Prophages in Different Hosts and Different Varieties of Citrus in Florida

Primers specific for each of the 4 abundant types were designed to enable the detection of the individual sequence types on different hosts of Las bacteria including citrus, periwinkle and psyllid. The specificity and sensitivity of primers for Type A, B, C and D were evaluated by conventional PCR using template DNA from pCR8/GW-TOPO plasmid with each of the 4 types of sequences obtained from screening the libraries. Due to the high sequence similarity between Type C and B, an optimized PCR protocol for Type C primer was developed to increase the specificity of Type C primer. Further study of the other 5 rare Types of sequence were not included in this study because of their very low abundance in the three clone libraries compared to Type A, B, C and D.

Using primers specific to each type, Las populations containing prophage Types A, B and C were confirmed in more than 99% of HLB-affected citrus and periwinkle, and Type D was detected from 94.6% and 100% of tested citrus and periwinkle plants, respectively (Table 4). Intriguingly, 75% of tested psyllid samples collected from greenhouse and field contained Type A, 74.5% contained Type B and C, but none of them contained Type D (Table 4), although most of the HLB-affected citrus plants in the field are Type D positive.

**Table 4:** Confirmation of four dominant prophage types of Las-infected plant and insect hosts.

Host	Total No. of samples	Real-time (Li)	Type A		Type B		Type C		Type D	
			No. of Pos.	%	No. of Pos.	%	No. of Pos.	%	No. of Pos.	%
Citrus	37	20.35–28.68	37	100	36	97.30	36	97.30	35	94.59
Periwinkle	42	17.21–23.41	42	100	42	100	42	100	42	100
Psyllid	129	17.14–29.72	97	75.19	93	72.09	88	68.22	5*	3.88

All these samples were weak positive with primers LJ860/863 only and were negative with other three sets of Type D specific primers including LJ759, LJ886/LJ861and LJ862/LJ827.

**Figure 4:**
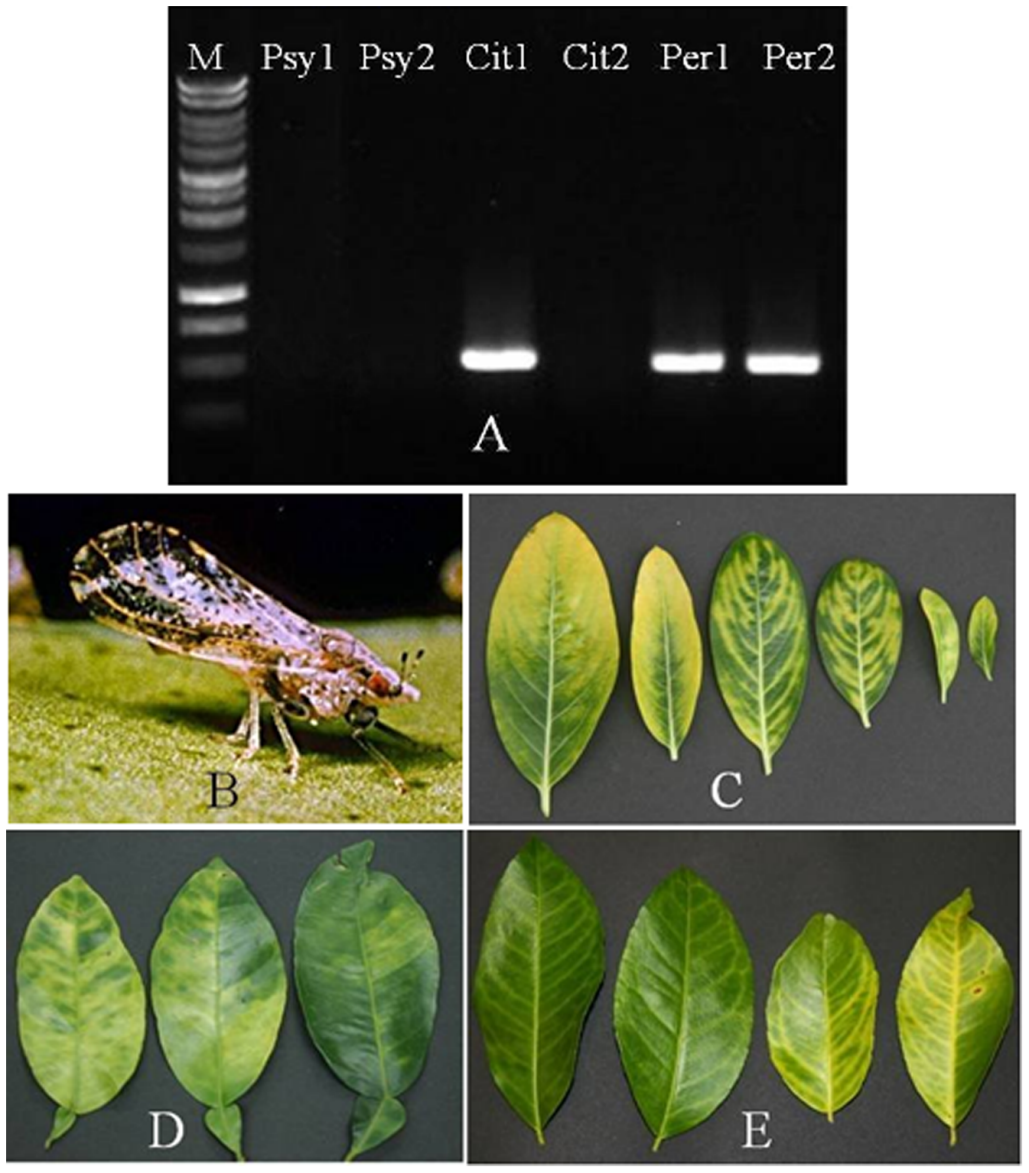
PCR amplification of Las Type D in different hosts using Type D specific primer. A: Gel image showing conventional PCR product from different hosts. Psy1/2 is psyllid DNA, Cit1/2 is citrus DNA, Per1/2 is periwinkle DNA; B: Single psyllid (ACP) used to prepare psyllid DNA; C: Symptomatic periwinkle leaves used to prepare DNA Per1 or Per2. D: Citrus leaves with typical blotchy mottle used to prepare DNA Cit1. E: Citrus leaves with vein yellowing symptoms used to prepare DNA Cit2.

Of 124 HLB-affected citrus samples collected from the USHRL farm in Fort Pierce, FL, 97.6% tested positive for Types A and B, 81.4% for Type C, and 86.3% for Type D. However, only 51.6% of the samples contained all four types. Compared to other cultivars, a lower percentage of sweet orange and grapefruit contained the Type C population, and a lower percentage of grapefruit and pomelo contained the Type D population (Table 5). Although no direct correlations were observed between Types A, B and C and HLB symptoms, all DNA samples that did not contain Type D in this test were from infected citrus leaves without typical blotchy mottle symptom (Figure 4).

**Table 5:** Typing analysis for 124 *Candidatus* Liberibacter asiaticus (Las)-infected citrus samples collected from 100 citrus plants at the USHRL’s Ft. Pierce farm in Fort Pierce, FL.

	Total No. of samples	A		B		C		D		A–D*
		No. of Pos.	%	No. of Pos.	%	No. of Pos.	%	No. of Pos.	%	
Grapefruit	8	8	100	8	100	2	**25**	5	**62.5**	**12.5**
Sweet orange	31	31	100	31	100	20	**64.5**	28	90.3	54.8
Sour orange	11	11	100	9	81.8	9	81.8	10	90.9	54.5
pomelo	9	9	100	9	100	9	100	5	**55.6**	55.6
Tangelo	14	13	92.9	14	100	14	100	12	85.7	85.7
Mandarin	37	36	97.3	37	100	34	91.9	34	91.9	62.1
Lemon	14	13	92.9	13	92.9	13	92.9	13	92.9	78.6
**Total**	**124**	**121**	**97.6**	**121**	**97.6**	**101**	**81.4**	**107**	**86.3**	**51.6**

–D represents the number of samples with all four types including Type A, B, C and D. A

### Type Variations of Las Prophages in Different Citrus Samples from Different Geographic Origins

All four sequence types were found in citrus samples from only five of the 16 counties in Central and South Florida from which 91 citrus samples were collected (Table 6). A low percentage of the Type D population (less than 20%) was observed in Las-infected citrus samples from Hillsborough, Manatee and Polk counties. These are neighboring counties located in west central Florida (Figure S1). Interestingly, two samples, one from Collier and the other from Martin County contained none of the four sequence types although the Ct values were 22.14 and 20.52, respectively, by qPCR using primers and probe targeting the Las 16S rRNA gene.

**Table 6:** Typing analysis for 91 citrus samples from 16 counties in Florida.

	Total No.of samples	A		B		C		D		A–D^&^
		No. of Pos.	%	No. of Pos.	%	No. of Pos.	%	No. of Pos.	%	
Brevard	5	4	80	4	80	4	80	3	60	60
Charlotte∧	5	5	100	5	100	5	100	5	100	100
Collier#	6	5	83.3	5	83.3	4	66.7	3	50	33.3
Desoto∧	6	6	100	6	100	6	100	6	100	100
Hardee∧	7	7	100	7	100	7	100	7	100	100
Indian River	6	6	100	6	100	5	83.3	5	83.3	33.3
Highlands	9	8	88.9	9	100	8	88.9	3	33.3	22.2
Hillsborough*	4	4	100	4	100	4	100	0	0	0
Hendry	6	4	66.7	6	100	3	50	3	50	33.3
Okeechobee	5	5	100	5	100	4	80	4	80	60
Martin#	6	5	83.3	5	83.3	5	100	5	83.3	66.7
Manatee*	4	4	100	4	100	4	100	0	0	0
Polk*	10	10	100	9	90	9	90	2	20	10
St. Lucie∧	5	5	100	5	100	5	100	5	100	100
Osceola	5	5	100	5	100	3	60	3	60	40
Glades∧	2	2	100	2	100	2	100	2	100	100
**Total**	**91**	**85**	**93.4**	**87**	**95.6**	**78**	**85.7**	**56**	**61.5**	**53.8**

^#^ One sample from Collier and the other one from Martin county tested negative for all four types;

Samples from these three counties contained a low percentage of Type D;

Samples from these counties containing Las populations with all four types of sequences.

–D represents the number of samples with all four types including Type A, B, C and D. & A

Twelve Las-infected citrus or psyllid DNA samples, both with and without the *hyv*_I_ and *hyv*_II_ genes (Table S1), were collected from China, India, the Philippines, Thailand or Brazil and were subsequently used for typing analyses with primers specific for the Floridian Types A, B, C and D sequences. Based on our previously findings, the *hyv*_I_ was located in FP1 and the *hyv*_II_ was located in FP2 [18]. However, the citrus isolates from China, India and Brazil did not have Type A even some of them contained the *hyv*_I_ gene; Type B was only detected in a single Chinese citrus sample along with the *hyv*_II_ gene; Type C was detected in citrus samples from China, Thailand and Brazil; and Type D was only detected in one Chinese and one Thai samples (Table S1). These preliminary typing results indicate that the prophage sequence variations present in the Las isolates can be found not only in different hosts and different citrus varieties within the same location but also in geographically distinct regions.

### Incomplete Phage/prophage 3 (iFP3) Derived from the Recombination of FP1 and FP2

**Figure 5:**
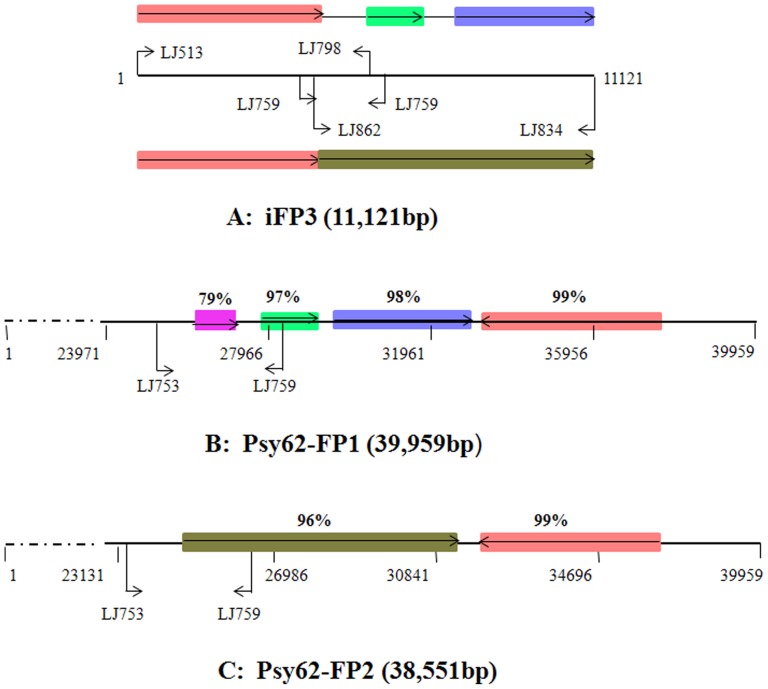
Schematic diagram indicating the rearrangements of iFP3 in comparison with FP1 and FP2. A: Extension of the Type D region (iFP3) by cloning and sequencing of long PCR products using primer sets LJ513/LJ798 and LJ862/LJ834 from Las-infected periwinkle. B: iFP3 homologous sequences in prophage FP1; C: iFP3 homologous sequences in prophage FP2. Rectangles in different colors represent the homologous sequences; arrow inside of each rectangle represents the orientation of that sequence. The percentages in B and C represent the sequence similarities compared to the same color sequences in A.

The 2,123 bp Type D sequence was obtained from a PCR product amplified by the inverted repeat primer LJ759 (Table 2). Sequence analysis implied that Type D was the result of a recombination or rearrangement event in the prophage regions. Based on the flanking sequences of the Type D region, two pairs of primers, LJ513/LJ798 and LJ862/LJ834 (Figure 5A), were used to perform sequence extension of the Type D region from Las-infected periwinkle. After cloning and sequencing these two overlapping PCR products, an 11,121 bp region was assembled. The extended Type D sequence was tentatively named incomplete FP3 (iFP3). A comparison of the iFP3 sequence with prophage FP1 and FP2 was performed after confirmation of this 11,121 bp region in three different Las isolates from periwinkle by long PCR using primers LJ513/LJ834 (Figure S2). Sequence analysis results indicate iFP3 was derived from the recombination of prophage FP1 and FP2. The three rearranged fragments in iFP3 share 97–99% similarity with the homologous region in psy62-FP1 (Figure 5B). In a comparison of the iFP3 sequence with psy62-FP2, the two rearranged fragments shared 96% and 99% similarity with the corresponding homologous region in psy62-FP2 (Figure 5C). Seventeen ORFs were predicted from iFP3 as listed in Table S2. Among them, half were annotated as phage-related proteins and the other half were hypothetical proteins. They share a higher similarity with the proteins in FP2 than FP1, and only five ORFs showed homology to corresponding proteins in both FP1 and FP2. These include a truncated gene due to deletion (Table S2).

## Discussion

As one of the most dynamic components in bacteria genome, prophages have an enormous influence on host evolution and intraspecies diversity [43]. In his study, nine types of sequences were identified from the homologous regions of Las prophages in FP1 and FP2, indicating a mixed population of Las bacteria amongst Florida Las isolates. Extensive homologous recombination, and deletion or insertion events between FP1 and FP2 resulted in changes to the population dynamics within different hosts or different isolates. Further evidence of frequent homologous recombination events in the Las prophages was also observed by sequencing PCR products amplified with primers designed from the conserved region within the Types A–C sequences (primer sets LJ825/LJ826 and LJ825/LJ1253 in Table S3). Three additional sequence Types F (1,866 bp, accession number: JX275498), G (1,573 bp, accession number: JX275499) and H (2,355 bp, accession number: JX275500), were identified from Las-infected periwinkle and were all confirmed as low abundance in plant hosts only (data not shown).

Among the four abundant types of sequences identified from the three libraries, the Las populations containing the Type D sequence drew the most attention because this new sequence type appeared to be associated with disease symptoms on citrus plants. It also implies the insect transmission determinants maybe present in the prophages since type D, a recombinant of type A and B, cannot be transmitted by the psyllids (Figure 4). In an effort to validate the absence of the Type D population in psyllids, other three sets of Type D specific primers LJ759, LJ886/LJ861 and LJ862/LJ862 (Table 1, Table S3) were used to amply the Type D region from the five psyllid samples with weak positive amplified by primers LJ860/LJ863 (Table 4). PCR results yielded all negative from 5 psyllid samples, in contrast, these primers yielded positive amplifications from the infected plant samples (data not shown). We hypothesize that the Las population containing Type D may not be able to multiply in a psyllid. The faint amplification bands seen in these Las-infected psyllids using primers LJ860/863 may come from degraded Las DNA that was taken up by the psyllids. It is also possible that these amplicons represent another variants of the Type D. It is of interest to note that we were able to detect high titers of Type D population in stripe mealybugs, a new insect host of Las bacterial [44], although the Type D Las bacteria were not able to cause disease despite their ability to be transmitted to periwinkle plants (Duan et al., unpublished data). All these together imply that the Las prophages may play a role in insect transmission in addition to disease development.

Primers specific to each abundant type were designed in order to differentiate the Las populations amongst the various hosts in Florida. It is worth noting that two citrus samples, one from Collier county and the other one from Martin county, tested negative by all four primer sets specific to Types A, B, C and D even though the samples contained high titers of Las bacteria by real-time PCR using 16S rRNA gene-based primers and probe. Further evidence of the high titer of Las bacteria in these samples was confirmed by PCR using an additional four primer sets, which targeted the Las genome outside the rRNA operon and prophage regions (Figure S3). These results suggest that the Las bacteria in these two samples may either lack the known prophage/phage in the genome or contain very different sequences in this prophage region. Similar typing results were also observed the Las global isolates, particularly in the Las Thai isolates. Using same primer LJ753/759, at least eight types of sequences were identified from the Tai isolates (Duan et al., unpublished data). Similar to the variations in the Florida isolates, the homologous recombination, deletion or insertion events were also observed among the Thai isolates. It is important to note that none of the sequence types of the Thai isolates was identical to any sequence types of the Florida Las isolates. All these result not only confirmed the mix populations in the Thai isolates, but also inferred the dramatic sequence variations in prophage region within the Las global isolates.

The HVRs named as Type A and B contained two homologous gene clusters within the FP1 and FP2 prophages of the Las psy62 genome. One partial and three full ORFs were predicted in Types A, B and their derivatives. The partial ORF encodes the small subunit of a putative phage terminase. Of the three full ORFs, two were annotated as hypothetical proteins, and the other one was annotated as a putative transcriptional regulator belonging to the XRE family. However, these proteins shares 70% similarity between the ones encoded in the Types A and B, and 100% identical in the ones encoded in the Types B, C, and D. Interestingly, a large intergenic region (IGR) was observed consisting of 1,104 bp in Type A and 1,351 bp in Type B. This IGR is located between two unidirectionally transcribed coding sequences that encode a putative phage terminase small subunit and a transcriptional regulator. Intergenic regions of bacterial chromosomes have been suggested to carry important functional units, such as transposable elements [45], or act as small regulatory RNAs or transcribed RNAs that regulate transcription and gene expression [46], [47]. Sequence analyses revealed that large amounts of the direct or inverted repeats and an AT-rich palindromic sequence were observed in this IGR, and the special structure formed by the repeat or the AT-rich palindromic sequence may play an important role in the instability of the prokaryotic and eukaryotic genomes [48], [49]. Using the UNAFold program in the RNAstructure software [50], both DNA and RNA secondary structures from the large IGR in Types A and B were predicted, and a more complicated stem-loop structure (SLS) was observed from the IGR in Type B than in Type A. The DNA secondary structure from the IGR in Type B presented a long chain SLS with a stem longer than 50 bp at the beginning of the chain (Figure S4). This longer stem in the SLS has been reported to affect RNA stability and functionality due to the formation of secondary structure complex [51]. Type B is the dominant type in Las plant hosts and one of the abundant types in Las-infected psyllids (Table 2). A schematic diagram in Figure 2 indicates that the Types C, C1 and C2 were all derived from Type B by deletion or insertion events. Since the SLS plays an important role in DNA replication and recombination [52], Types C, C1 and C2 may be a direct result of the influence of the SLS region on replication. Furthermore, during Sanger sequencing for all the Type B clones, we encountered a sudden stop at nucleotide 388, which inferred a strong secondary structure in the DNA template.

Extension of the Type D sequence revealed the presence of a third prophage/phage in the Las genome in addition to FP1 and FP2. Annotation of iFP3 implied that the recombination or rearrangement events resulted in less similarity of the proteins (3 out of 17 ORFs) when compared to the corresponding proteins in FPI and FP2, respectively, which may yield different functions (Table S2). Since the iFP3 is abundant in different host plants and is the only prophage associated with the blotchy mottle symptom, the presence of iFP3 appears to correlate with the disease development. More importantly, the level of iFP3 was either extremely low or absent in Las-infected psyllids, indicating that the Las cells containing iFP3 may not survive or be able to replicate in the psyllids in contrast to those in Las-infected mealybugs. Further investigation is needed to shed light on how the dynamics of Las populations affect insect transmission and HLB disease development, and thereby may yield new control strategies for citrus HLB.

## Supporting Information

Figure S1 **Florida county map with the HLB sampling sites from central to south Florida.** Counties with diagonal hatching were sampling locations, and the county names are listed on the left of the map.
(TIF)

Figure S2 **Confirmation of extended 11,121 bp type D region sequence by long PCR using primer set LJ513/LJ834.** M is 1 kb DNA ladder from Promega; lanes 1–3 are DNA extracted from three Las-infected periwinkle plants, PP11, PP15 and P1, respectively.
(TIF)

Figure S3 **PCR amplicons of the outer member protein, β-operon region and tufB gene region, respectively.** M is 1 kb DNA ladder from Promega; lanes 1–2 are DNA samples from Collier and Martin county in Florida with Ct. value 22.14 and 20.52, respectively by 16S rRNA gene based on TagMan real-time PCR primers and probe [32].
(TIF)

Figure S4 **DNA secondary structure of the Type A and B intergenic regions.** The sequences of ‘*Candidatus* Liberibacter asiaticus’ Type A and B intergenic regions were predicted by RNAstructure software.
(TIF)

Table S1 Typing analysis for the Candidatus Liberibacter asiaticus isolates from China, India, the Philippines, Thailand and Brazil.
(DOCX)

Table S2 List of ORFs predicted from the incomplete prophage 3 (iFP3) of Candidatus Liberibacter asiaticus.
(DOCX)

Table S3 Primers used for identification and confirmation of Candidatus Liberibacter asiaticus Types F, G, and H.
(DOCX)
