# Supplementary figures and images for: Prophage-Mediated Dynamics of ‘Candidatus Liberibacter asiaticus’ Populations, the Destructive Bacterial Pathogens of Citrus Huanglongbing
Source: PLoS One. 2013 Dec 13;8(12):e82248. doi: 10.1371/journal.pone.0082248 (PMC3862640; doi:10.1371/journal.pone.0082248)

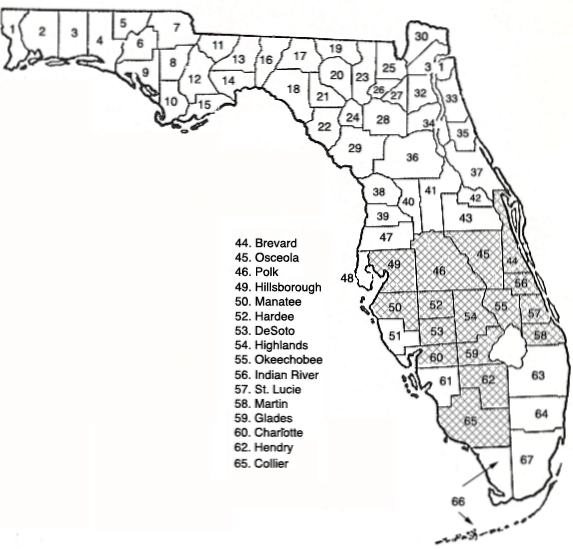

Supplement: Figure S1 — Florida county map with the HLB sampling sites from central to south Florida. Counties with diagonal hatching were sampling locations, and the county names are listed on the left of the map. (TIF) [file pone.0082248.s001.tif]

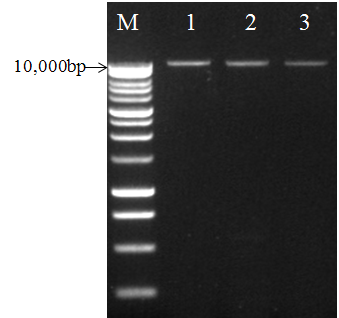

Supplement: Figure S2 — Confirmation of extended 11,121 bp type D region sequence by long PCR using primer set LJ513/LJ834. M is 1 kb DNA ladder from Promega; lanes 1–3 are DNA extracted from three Las-infected periwinkle plants, PP11, PP15 and P1, respectively. (TIF) [file pone.0082248.s002.tif]

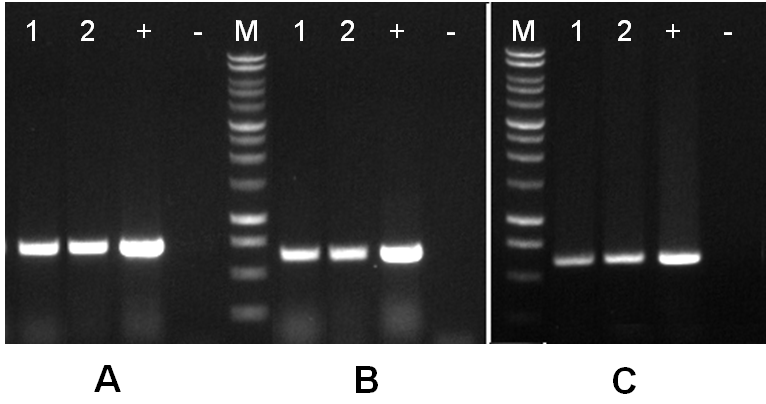

Supplement: Figure S3 — PCR amplicons of the outer member protein, β-operon region and tufB gene region, respectively. M is 1 kb DNA ladder from Promega; lanes 1–2 are DNA samples from Collier and Martin county in Florida with Ct. value 22.14 and 20.52, respectively by 16S rRNA gene based on TagMan real-time PCR primers and probe [32]. (TIF) [file pone.0082248.s003.tif]

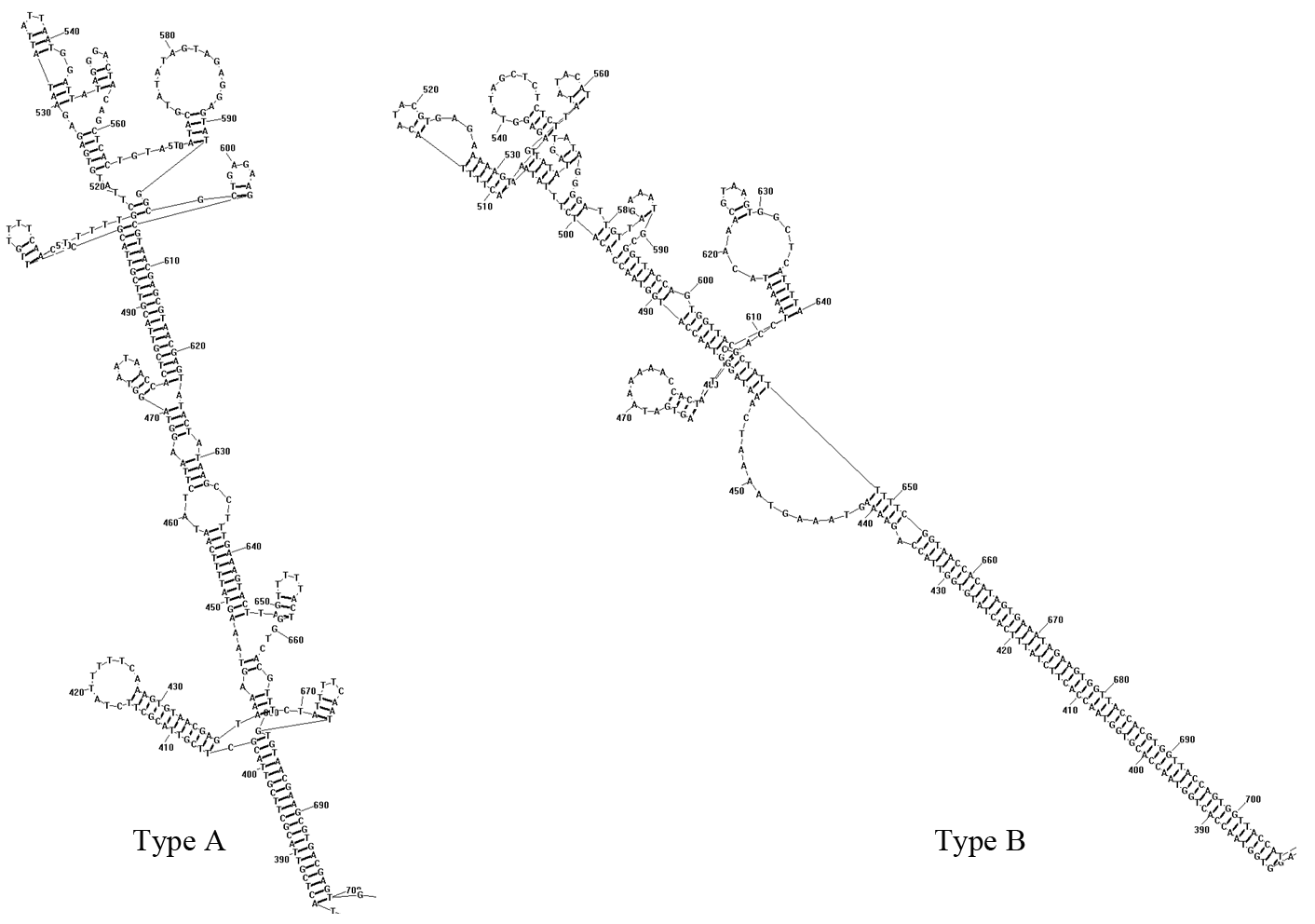

Supplement: Figure S4 — DNA secondary structure of the Type A and B intergenic regions. The sequences of ‘Candidatus Liberibacter asiaticus’ Type A and B intergenic regions were predicted by RNAstructure software. (TIF) [file pone.0082248.s004.tif]
