# Supplementary material for: Prophage-Mediated Dynamics of ‘Candidatus Liberibacter asiaticus’ Populations, the Destructive Bacterial Pathogens of Citrus Huanglongbing
Source: PLoS One. 2013 Dec 13;8(12):e82248. doi: 10.1371/journal.pone.0082248 (PMC3862640; doi:10.1371/journal.pone.0082248)
Supplement: Table S1 — Typing analysis for the Candidatus Liberibacter asiaticus isolates from China, India, the Philippines, Thailand and Brazil. (DOCX) [file pone.0082248.s005.docx]

**Table S1. Typing analysis for the *Candidatus* Liberibacter asiaticus isolates from China, India, the Philippines, Thailand and Brazil.**

| **Sample name** | **Ct.-Li** | ***hyv*_I_** | ***hyv*_II_** | **A** | **B** | **C** | **D** |
| --- | --- | --- | --- | --- | --- | --- | --- |
| CHA-C2 | 25.68 | - | ++ | - | ++ | + | - |
| CHA-C17 | 26.81 | + | - | - | - | ++ | + |
| CHA-Psy-1 | 26.77 | + | - | - | - | - | - |
| IND-C11 | 24.61 | - | - | - | - | - | - |
| IND-C15 | 24.71 | - | - | - | - | - | - |
| IND-Psy-1 | 29.06 | - | - | + | - | - | - |
| IND-Psy-2 | 24.66 | + | - | ++ | - | - | - |
| PHI-Psy-5 | 18.67 | + | - | ++ | - | - | - |
| PHI-Psy-25 | 19.37 | - | + | ++ | - | - | - |
| THA-C1 | 24.06 | ++ | - | ++ | - | ++ | - |
| THA-C2 | 21.22 | - | ++ | ++ | - | ++ | ++ |
| BRA-C1 | 23.96 | ++ | - | - | - | ++ | - |

‘++’ indicates strong positive; ‘+’ indicates weak positive; ‘-’ indicates negative
