# Supplementary material for: Prophage-Mediated Dynamics of ‘Candidatus Liberibacter asiaticus’ Populations, the Destructive Bacterial Pathogens of Citrus Huanglongbing
Source: PLoS One. 2013 Dec 13;8(12):e82248. doi: 10.1371/journal.pone.0082248 (PMC3862640; doi:10.1371/journal.pone.0082248)
Supplement: Table S2 — List of ORFs predicted from the incomplete prophage 3 (iFP3) of Candidatus Liberibacter asiaticus. (DOCX) [file pone.0082248.s006.docx]

**Table S2. List of ORFs predicted from the incomplete prophage 3 (iFP3) of *Candidatus* Liberibacter asiaticus.**

| **iFP3** | **Las psy62 genome** | | **Las psy62 prophage FP2** | | **Product** |
| --- | --- | --- | --- | --- | --- |
|  | **Gene locus** | **Similarity*** | **Gene locus** | **Similarity*** |  |
| TD_001 | CLIBASIA_00030 | 100% | gm_215 | 100% | putative DNA polymerase from bacteriophage origin |
| TD_002 | CLIBASIA_00025 | 99.5% | gm_210 | 99.5% | Phage-related protein |
| TD_003 | CLIBASIA_00020 | 100% | gm_205 | 100% | Prophage antirepressor |
| TD_004^#^ | CLIBASIA_00015 | 100% | gm_200 | 100% | Conserved hypothetical protein |
| TD_005^#^ | CLIBASIA_00015 | 95.2% | gm_200 | 95.2% | Hypothetical protein |
| TD_006 | CLIBASIA_00010 | 100% | gm_195 | 100% | Hypothetical protein |
| TD_007 | CLIBASIA_00005 | 98.4% | gm_190 | 100% | Hypothetical protein |
| TD_010 | CLIBASIA_05625 | 70.3% | gm_130 | 100% | Putative transcriptional regulator |
| TD_015 | CLIBASIA_05630 | 78.1% | gm_135^#^ | 100% | Hypothetical protein |
| TD_020 | CLIBASIA_05635 | 100% | gm_140 | 100% | Conserved hypothetical protein |
| TD_025 | CLIBASIA_05640 | 100% | gm_145^#^ | 100% | putative phage-related protein |
| TD_030 | CLIBASIA_05645 | 100% | gm_150 | 100% | putative phage-related protein |
| TD_035 | CLIBASIA_05650^#^ | 82.1% | gm_155 | 99.3% | putative phage-related protein |
| TD_040 | CLIBASIA_05655^#^ | 80.8% | gm_165 | 100% | Hypothetical protein |
| TD_045 | CLIBASIA_05660 | 97.5% | gm_170 | 84.6% | P4 family phage/plasmid primase |
| TD_050 | CLIBASIA_05665 | 96.9% | gm_175 | 98.4% | Conserved hypothetical protein |
| TD_055 | CLIBASIA_05670 | 98.5% | gm_180 | 100% | Hypothetical protein |

*protein similarity compared to the corresponding proteins in Type D.

^#^ truncated gene due to deletion/insertion during recombination.
