# Supplementary material for: Prophage-Mediated Dynamics of ‘Candidatus Liberibacter asiaticus’ Populations, the Destructive Bacterial Pathogens of Citrus Huanglongbing
Source: PLoS One. 2013 Dec 13;8(12):e82248. doi: 10.1371/journal.pone.0082248 (PMC3862640; doi:10.1371/journal.pone.0082248)
Supplement: Table S3 — Primers used for identification and confirmation of Candidatus Liberibacter asiaticus Types F, G, and H. (DOCX) [file pone.0082248.s007.docx]

**Table S3. Primers used for identification and confirmation of *Candidatus* Liberibacter asiaticus Types F, G, and H**

| **Name** | **Sequence (5’-3’)** | **Description** |
| --- | --- | --- |
| LJ825 | TCAGCCACTTTGGGGTAGCAG | Used for identification of Types F, G, and H |
| LJ826 | TTACAGCACTTAAAAACTCGTAG | Used for identification of Type H with LJ825 |
| LJ1253 | AAAGGGTGGTTGTAGGTTAAG | Used for identification of Types F and G with LJ825 |
| LJ886 | GGGCTTCATCTAATAACCTCC | Type D specific when using with LJ861 |
| LJ861 | TCTAAAATGGTGGAATCAGACC | Type D specific when using with LJ886 |
| LJ827 | CAACACCTAAATGAAAAAATACTG | Type D specific when using with LJ862 |
